# Publishing controversy

**DOI:** 10.1192/bjb.2020.127

**Published:** 2021-10

**Authors:** Norman A. Poole

**Affiliations:** South West London and St George's Mental Health NHS Trust, London, UK

**Keywords:** Editorial policy, publishing ethics, gender identity, gender incongruence

## Abstract

Two recent papers on a controversial topic in this journal attracted significant criticism from readers. This editorial addresses these criticisms and describes changes to be made to the journal's editorial and review procedures in light of the complaints received.

Psychiatry, like other branches of medicine, is no stranger to controversy. Anthony Clare's *Psychiatry in Dissent*^1^ ran the gamut of contested areas – from the validity of psychiatric diagnosis to electroconvulsive therapy and psychosurgery – which are, to varying degrees, still with us. Some have argued that disputes over the concept of mental disorder generally^2^ and certain specific categories^3^ merely demonstrate that psychiatry is a pseudoscience: psychiatric diagnosis, unlike the remainder of medicine, is a matter of value judgements rather than ‘hard facts’. In this view, psychiatrists are really just pathologising people who transgress some sort of social norm.

Since the heyday of such arguments, it has been increasingly understood that, yes, values are involved in diagnosis, but this is true also in other medical specialties. In Peter Sedgwick's memorable phrase, ‘The fracture of a septuagenarian's femur has, within the world of nature, no more significance than the snapping of an autumn leaf from its twig’.^4^ But we deprecate the first, so consider it disordered. Yet psychiatry remains the more controversial specialty. Bill Fulford draws an analogy between the different ways we use the word ‘good’ when thinking about a ‘good strawberry’ versus a ‘good painting’.^5^ It turns out that there is greater agreement about the former than the latter, hence less conflict and controversy. And what constitutes a person's very nature and identity is a lot more like paintings than strawberries.

The *BJPsych Bulletin*, representing views within and about psychiatry, cannot evade controversial issues, but neither should we court them for their own sake. At the heart of such controversies are real people with real lives, often ostracised and denigrated. So, we have a duty to be respectful and balanced when articles on controversial topics are accepted for publication. We recently published two papers^6^ on gender incongruence that have attracted a significant number of letters and complaints, particularly regarding Marcus Evans’ opinion piece ‘Freedom to think: the need for thorough assessment and treatment of gender dysphoric children’.^7^ In light of the criticisms, we reviewed the article and have published a corrigendum of clarifications and additional information that provides a stronger evidence base for his arguments. Importantly, Evans has also provided a declaration of interest statement addressing his involvement in a judicial review of gender-affirming treatment for minors.

## Changes to editorial procedures

Criticisms of the paper have been discussed by the editorial board. It was never the intention for the board to review the evidence for and against gender-affirming treatment. We appreciate that there are gaps in the evidence base concerning psychological outcomes of gender-affirming surgery,^8^ so see the journal's role as enabling discussion. We seek to present the suffering caused by prejudice and failings in care systems, address omissions in the evidence base, and enable clinicians and patients to express concerns about ethical practice. The journal's position is not to censor one or other argument – albeit clarity and care are needed when discussing emotive issues and the potential harms of psychiatric practice. The editorial board have discussed how handling editors should deal with submissions about such controversial topics, and agreed the following recommendations.

- The Special Articles category currently combines both review and opinion pieces. We will reintroduce Review and Opinion type papers to clarify for readers the nature of the content.
- We have added to the instructions for authors that Opinion pieces can include references from news items and blogs.
- We will not accept an Opinion article with reviews solely by the author's suggested reviewers. As a general rule, editors do avoid this. However, it is now a firm policy for Opinion pieces to ensure that an independent review is always sought, even though this may lead to delays to the peer review process. We may call on editorial board members as required to provide reviews.
- Reviewer invitation templates will be revised to include a link to the COPE Ethical Guidelines for Peer Reviewers and the Reviewer Support Hub on Cambridge Core (in development). We will encourage reviewers and editors to use sites such as https://mediabiasfactcheck.com/ to check the level of bias of non-scholarly sites.
- If an article involves a controversial issue, handling editors will seek to balance it, either in reviews or with a counterbalancing article, commentary or eLetter, although we acknowledge that this may not always be possible.

I appreciate that the published corrigendum and review of editorial processes will, for some, not go far enough. Many of the complainants sought retraction rather than correction and pointed to the distress such papers can cause an already marginalised group of people. I am deeply sorry for the hurt caused and have invited authors of the complaint letters to submit counterbalancing articles and/or eLetters to ensure that the spectrum of opinions is presented. Readers are welcome to submit correspondence by clicking the e-letters tab when accessing the article via the following link: https://doi.org/10.1192/bjb.2020.72.

COPE, the Committee on Publication Ethics, has guidelines^9^ for editors considering retraction of an academic paper. Its criteria cover situations where there is clear evidence of unreliability or falsification of data, plagiarism, copyright infringement or manipulation of the peer review process. These do not apply here. Failure to disclose a major conflict of interest can also lead to retraction where non-disclosure has ‘unduly affected interpretations of the work’ by editors and peer reviewers. However, Evans has been candid about his opinions, which are of a piece with his involvement in the judicial review.

Derek Bolton's *What is Mental Disorder?*^10^ has long struck me as a fine argument for the constructive value of disagreement. Where concepts are contended, they are subject to competing pressures from the various stakeholders, including patients, carers, doctors, psychologists, social scientists, the general public and politicians. He was referring to disputes about the boundary between order and disorder, health and illness, but it applies to controversial issues within psychiatry generally. In this spirit, the *BJPsych Bulletin* will always strive for open, transparent and respectful dialogue.

## About the author

**Norman A. Poole** is Editor of the *BJPsych Bulletin* and a consultant neuropsychiatrist at St George's Hospital, South West London and St George's Mental Health NHS Trust, UK.
